# The Public and Doctors for the Army

**Published:** 1915-08-28

**Authors:** 


### THE PUBLIC AND DOCTORS FOR THE ARMY.

It is evident that a very serious strain is to be placed on the resources of the medical profession by the demands of the Army. Practically every doctor of military age has been told that his country needs him. This is a very serious matter for the doctors, and a very serious matter also for the public. Many of the former will have to make great sacrifices, and it is for the public to see that the burden is not unnecessarily increased. They must do something to co-operate with the profession at a time when everything has to yield to the imperative claims of national safety. Victory must be fought for, and hardness endured, at home as well as in the trenches.

As the doctors who remain for civil practice will be relatively few, they will be under an added burden of work. To an increasing extent they will be called on to act for their absent colleagues, and in not a few instances their work will be doubled. Further, there is a plain intimation that the authorities not only want doctors for the armies in the field, but that ere long they will need much more help at home. This means that doctors in civil practice will be more and more needed to give service to wounded soldiers in hospitals. Here, again, therefore, the civilian practitioner will have added duties. There is a limit to human endurance, and it is a certain prospect that this will be reached, or even passed, unless the public recognise widely the new conditions under which the profession has to work. The appeal has been made before, but it must be repeated. Serious illness in the house is so urgent a position that nothing else seems of importance. Yet here, as well as in less acute positions, the time and endurance of the practitioner will have to be considered. Unnecessary demands, unreasonable hours, multiplied calls, trivial complaints—all these in the circumstances must be classed as anti-social sins. The designation is justified, for proceedings of this order, tending as they do to exhaust a practitioner's capacity and health, may deprive a whole district of medical advice. The brief position is that the British public is going to have comparatively few doctors at its call, and it will be wise to take care of them.

Sooner or later the men who go on active service will come home again. It is a small thing to ask that their patients shall prove loyal to them. In their absence they will have been represented by a colleague who will be anxious to show that he has been careful of their interests. The patients themselves must co-operate to this end. Considerations of personal preference, often purely fanciful, readily arise in medical practice, and to some persons a new friend, at least for a time, ever seems better than an old one. We should be the last to suggest that a doctor has any property in his patients, but he certainly has the right to expect gratitude for past services, and a loyal welcome when he returns after time spent in the service of his country. It would be a poor recognition to find that those who had benefited by his skill and kindliness had readily forgotten both the one and the other. The times are teaching the need for sacrifice; they are teaching also the need for mutual consideration. At this moment the military authorities are telling the profession that the need for doctors is both urgent and extreme. Let the public see that the answer to this call is recognised as a display of practical patriotism, and that it involves no loss which may be avoided by a little thoughtfulness, loyalty, and good will.

